# The biological role of N-acyl-homoserine lactone-based quorum sensing (QS) in EPS production and microbial community assembly during anaerobic granulation process

**DOI:** 10.1038/s41598-018-34183-3

**Published:** 2018-10-25

**Authors:** Haijun Ma, Sijia Ma, Haidong Hu, Lili Ding, Hongqiang Ren

**Affiliations:** State Key Laboratory of Pollution Control and Resource Reuse, School of the Environment, Nanjing University, Nanjing, 210023 Jiangsu P. R. China

## Abstract

Although N-acyl-L-homoserine lactone (AHL) based quorum sensing (QS) phenomenon has been observed in mature anaerobic granules, the biological role of AHL-based QS system in anaerobic granulation process remains unexplored. For the first time, a long-term anaerobic bioreactor was operated for 168 days to investigate the biological role of AHL in the granulation process which was divided into three phases (phase I: floccular, phase II: granulation, phase III: maturation). Two different AHLs including C8-HSL and C10-HSL were characterized at nanogram levels. The AHL level was elevated over 20-fold and strongly positively correlated with extracellular polymeric substances (EPS) production and sludge particle size during phase I-II. Exogenous addition of AHL to the floccular sludge also resulted in significantly increased EPS production. Metadata analysis suggested that the granulation process was accompanied by an increase in the abundance of QS-relevant microorganisms. The strong relationships (R > 0.9233, *p* < 0.01) among AHL concentrations, EPS (except loosely bound EPS), granulation and community variation indicated that AHL-mediated QS played an important role in coordinating community level behaviors associated with granulation, potentially through the regulation of EPS production and composition. This study gives a deep insight into the underlying QS-relevant mechanism of anaerobic granulation process.

## Introduction

In the past years, increasing attention has been paid to the quorum sensing (QS) in wastewater treatment systems such as membrane bioreactors (MBRs)^1,2^, sequencing batch reactors^3^, granular sludge^4^, and anaerobic ammonium oxidation (Anammox)^5,6^. QS, which is a communication strategy among microorganisms, is accomplished through the production and secretion of signal molecules, the accumulation of signal molecules in the intercellular environment and the regulation of downstream gene expression^7,8^. There have been several types of signaling molecules, among which N-acyl-L-homoserine lactone (AHL) is the most well-characterized QS signals. AHL-mediated QS system has been not only proved to be biologically active in microbial mats^9^, and the rumen^10^, but also ascertained to influence the biofilm formation in complex biofilm system^11^.

Recently, the studies have focused on the role of AHL-based QS signaling in granule formation. Granular sludge is generally considered to be a special form of biofilm. Feng, *et al*.^12^ verified the existence of AHL in mature anaerobic granules and concluded that the basic properties of granules were closely related to AHL levels. Our previous study reported that C8-homoserine lactone (C8-HSL) and C10-HSL were widely identified in all the collected anaerobic granules and 12 different microbes were speculated to be regulated by AHL to participate in the secretion of extracellular polymeric substances (EPS)^13^. These studies mainly investigated the diversity, distribution and function of AHL in mature anaerobic granules. Nevertheless, the role of AHL in anaerobic granule formation, especially in the organization of microbial population, remains unexplored. Investigating the biological function of AHL in anaerobic granule formation would enhance our knowledge about the underlying mechanism of granulation, hence contribute to the development of strategies to accelerate anaerobic granulation. Tan, *et al*.^14^ operated a sequencing batch reactor (SBR) as a model ecosystem to investigate how AHL affected the formation of aerobic granules. It was assumed that AHL was related to aerobic granulation through regulating EPS synthesis. EPS is generally believed to act as scaffold in granules, providing a protective three-dimensional matrix for microorganisms inside^15,16^. However, the role of AHL signals in the conversion from anaerobic floccular sludge to granular sludge is still not clarified. Moreover, as the sludge granulation is a consequence of self-immobilized microbial aggregation, investigating the biological impact of AHL-based QS on microbial community organization and intercellular cooperation during granulation period is of great importance for understanding the microbial foundation of anaerobic granulation process.

In this study, an up-flow anaerobic sludge blanket (UASB) reactor was operated as a model ecosystem for anaerobic granulation. All the key operational parameters, including temperature, pH, up-flow velocity, nutrient, trace elements and the transition from flocs to dense granules, were precisely controlled. The particle size, AHL concentrations, EPS, reactor performance, integrated with changes of microbial community through the granulation process were intensely monitored. By employing ecology study approaches to statistically analyze the acquired chemical and biological data, we explored the biological role of AHL signaling activity in the assembly and interaction of highly diverse community during the anaerobic granulation process.

## Materials and Methods

### The operation of bioreactor

**Figure 1:**
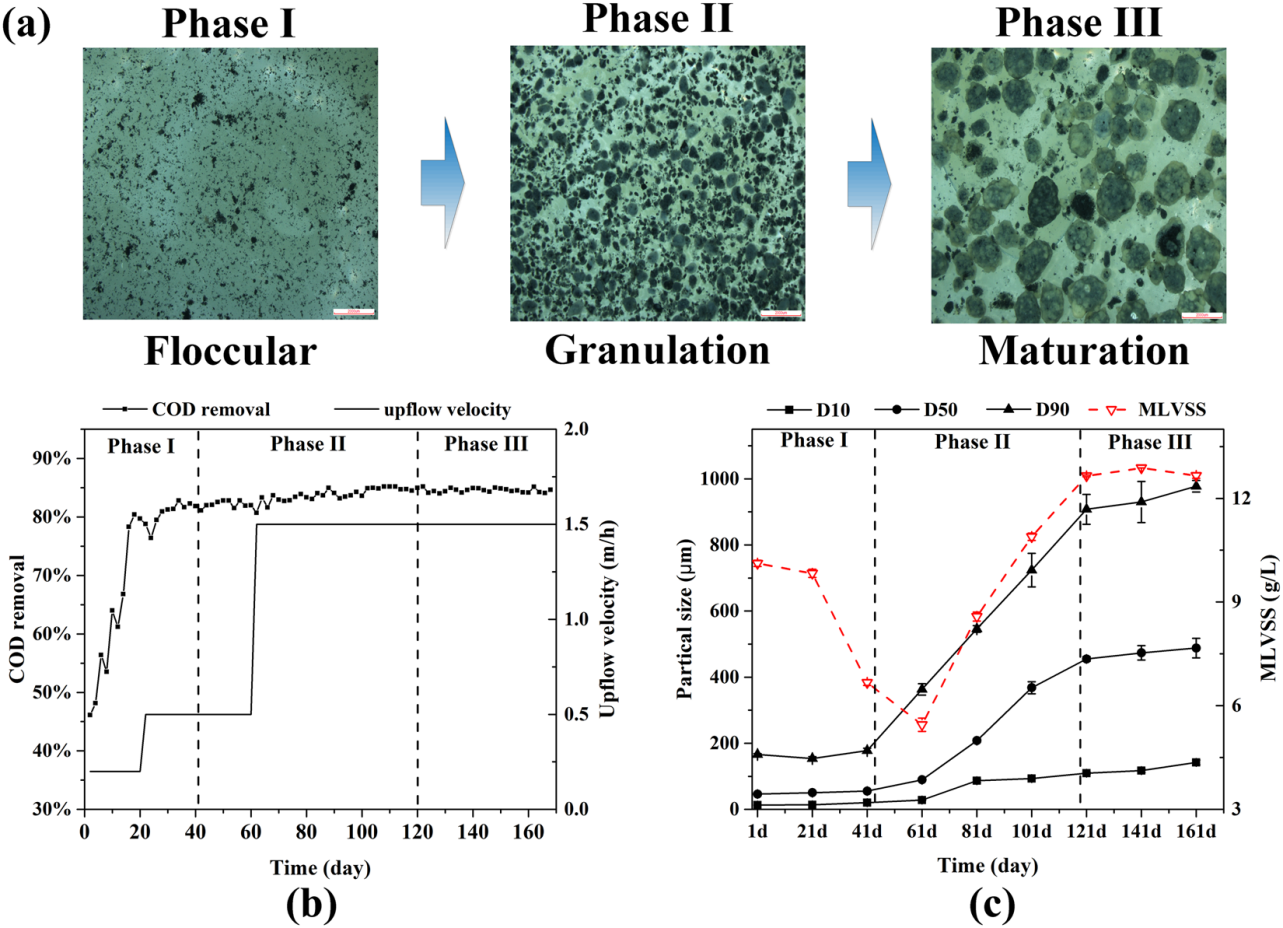
(**a**) Development of granular sludge in a UASB bioreactor based on microscopic visualization of structures, (**b**) COD removal and up-flow velocity and (**c**) sludge particle size distribution and MLVSS. Bar in (**a**) was 2000 μm. In (**c**), D10, D50 and D90 indicated that 10%, 50% and 90% of total particles were below the corresponding size distribution, respectively. The dotted line separates the operation period into three different phases.

A UASB bioreactor with a working volume of 2.55 L and a height/diameter ratio of 13.6 was operated for 168 days in continuous flow mode. The bioreactor was inoculated with 58.8% of floccular sludge collected from the anaerobic bioreactor of a pharmaceutical factory at Zhejiang, China. The mixed liquor initial volatile suspended solids (MLVSS) of the UASB bioreactor was 10.12 ± 0.09 g MLVSS/L and the seed sludge particle size was 46.2 ± 0.8 μm. The composition of the used synthetic wastewater was as follows (mg/L): glucose 3000, NaHCO_3_ 3000, NH_4_Cl 287, NaH_2_PO_4_·2H_2_O 75.5, CaCl_2_·2H_2_O 92 and 1.0 ml trace elements solution. The trace element solution contained (mg/L): FeCl_2_·4H_2_O 2000, MgSO_4_ 2000, MnCl_2_·4H2O 94.6, ZnCl_2_ 136, (NH_4_)_6_Mo_7_O_24_·4H_2_O 62.7, CoCl_2_·6H_2_O 110, CuCl_2_·2H_2_O 76, H_3_BO_3_ 60, NiCl_2_·6H_2_O 110, AlCl_3_·6H_2_O 108 and 1.0 ml 36% HCl. The temperature of the bioreactor was kept at 35 ± 1 °C using a circulating water pump (THS-15, Atpio Instrument Manufacutring Co., Ltd., Nanjing, China) and the pH was maintained at 7.1 ± 0.1. The up-flow velocity was controlled by pumping the effluent back into bioreactor at the bottom of the bioreactor. Different up-flow velocity was accomplished by changing the reflux ratio. Organic loading rate (OLR) was controlled by changing influent flow rate. Before day 20, the up-flow velocity and OLR was set at 0.2 m/h and 2 kg·COD/(m^3^·d). From day 21 to day 60, the up-flow velocity and OLR was set at 0.5 m/h and 3 kg·COD/(m^3^·d). From day 61 to day 168, the up-flow velocity and OLR was kept at 1.2 m/h and 4 kg·COD/(m^3^·d) (Fig. 1b,c).

The anaerobic granulation process was monitored every twenty days. Chemical oxygen demand (COD), MLVSS of anaerobic sludge was measured according to APHA standard methods^17^. The sludge particle size was determined by a laser particle size analysis system (Malvern Mastersizer 3000, Singapore). The sludge biomass sample was sampled and stored at −80 °C immediately for later DNA extraction.

### EPS analysis

The total EPS is comprised of loosely bound EPS (LB-EPS) and tightly bound (TB-EPS), which were extracted from the sludge biomass following a previous study^18^. Briefly, 5 mL sludge mixture was collected in a 15 mL centrifugal tube and centrifuged at 4000 × g for 2 min. The pellet was gently washed by 10 mL 1 × PBS buffer and the mixture was dewatered at 4000 × g for 2 min again. Then 10 mL 1 × PBS buffer was added into the centrifugal tube and the centrifugal tube was sonicated for 2 min before being shaked at 150 rpm for 15 min. The LB-EPS extraction solution was obtained by centrifuging the sludge mixture at 6000 × g for 10 min. Afterwards, 10 mL 1 × PBS buffer was added into the centrifugal tube again and the centrifugal tube was heated at 80 °C for 30 min in a water bath. The sludge mixture was shaked by hands every 2 minutes. The organic matter in the supernatant was considered to be TB-EPS after sludge mixture being centrifuged at 12000 × g for 5 min. The extracellular polysaccharides (PS) in extraction solution was measured using the phenol/sulphuric acid method^19^ and the extracellular proteins (PN) was measured using bicinchoninic acid assay method^20^.

### AHLs detection

A total of 13 different types of AHL standards were purchase as described before^13^. AHLs detection was conducted every 20 days. In brief, about 30 mL sludge sample was collected using a 50 mL centrifugal tube and centrifuged at 8000 × g for 2 min. Then the supernatant was replaced by 40 ml ethyl acetate and immediately sonicated in ice bath at 10 W/mL for 30 min (JY92-IID Broken Series, Xinzhi)^13^. Then the acetate extracts was obtained by centrifuging at 12000 × g for 2 min and was dried in nitrogen flow and resuspended in 800 μl methanol for qualitative and quantitative analysis later. The types and concentrations of AHLs in all the samples were analyzed using high performance liquid chromatography mass spectrometry (HPLC-MS/MS). The detecting method was described by Tan *et al*.^14^. Briefly, the flow rate was set at 0.2 mL/min. The solvent A was 25 mM ammonium formate solution with 0.1% formic acid and solvent B was prepared by dissolving 0.1% formic acid in methanol. The ration of solvent A to B was linearly changed from 60:40 to 5:95. Effluents were ionized in positive mode and detected by the multiple reaction monitoring approach^9,21^. Standard curves were established using mixed standard samples ranging from 1 μg/L to 200 μg/L. The AHLs extraction efficiency was measured by adding standard AHLs to heat-inactivated sludge biomass and calculating the recovery^22^.

### Add-back experiment

A total of about 180 ml of floccular sludge was collected from the bioreactor and equally distributed to 9 individual serum bottles. Each serum bottle was added with 80 mL synthetic wastewater. pH was kept around 7.0. C8-HSL or C10-HSL solution was prepared by dissolving C8-HSL or C10-HSL standards in ethanol. Afterwards, 80 μL C8-HSL solution was added to three serum bottles as replicates and 80 μL C10-HSL solution was added to another three serum bottles as replicates so that the final concentration of exogenous AHL was 5000 nM. 80 μL ethanol was added to the rest three serum bottles as negative control. The sludge mixture in all serum bottles was flushed by highly pure nitrogen gas to get rid of dissolved oxygen. EPS analysis was conducted immediately after the sludge was incubated for 24 h at 35 °C.

### 16S rRNA gene sequencing analysis of microbial community

For DNA extraction, the stored sludge sample was thawed at room temperature and centrifuged at 12000 × g for 2 min. Then the total DNA was extracted as described by Jiang, *et al*.^23^. After diluting each sample to 20 ng/μl using DNA-free water, V3V4 primers pairs were used to amplify each sample. Each PCR mixture consisted of 25 μl of 2 × PCR super mix (Trans, China), 2 μl of diluted DNA, 1 μl of 20 μM primer, and 21 μl ddH_2_O. The PCR reaction was carried out according our previous study^13^. EasyPure PCR Purification Kit (TransGen Biotech, China) was used to purify the PCR product. Then the amplicons of all the samples were sequenced on Miseq platform using 2 × 300 bp sequencing strategy. The sequence datasets were denoised and analyzed by Mothur (http://www.mothur.org/wiki/MiSeq_SOP)^24^. Detailed bioinformatics analysis was shown in the Supplementary Material.

### Statistical analysis

The Pearson correlation was calculated using SPSS (IBM, USA). The co-occurrence among microorganisms and AHL was visualized by Gephi (WebAtlas, France) using Reingold placement algorithm^25^. The abundance of microbe was shown as heatmap using R software with pheatmap package.

## Results and Discussion

### Development of anaerobic granule

To monitor the anaerobic granulation process, sludge biomass was sampled for physicochemical property and microbial community determination every 20 days. After 168 days of operation, mature anaerobic granules successfully formed in the UASB bioreactor. Primarily based on the mean particle size (50^th^ percentile distribution), the whole operation period was divided into three distinct phases, referred to as phase I, II and III (Fig. 1). The mean particle diameter of seed floccular sludge was 46 μm and the COD removal was only 46% at the beginning time. The mean particle size remained stable in phase I (days 1–41). Meanwhile, COD removal increased substantially to be around 80%. This was followed by the significant increase of mean particle size from 55 μm to about 455 μm during phase II (days 42–160). More and more microbial aggregates began to emerge and finally developed into mature compact spherical granules. Afterwards, the particle size of granules kept stable for the last 48 days (phase III, days 121–168). The COD removal of the bioreactor increased slightly to be around 84% as compared with that at the end of phase I. As for sludge biomass, MLVSS underwent an initial deduction and subsequent increasement. Intriguingly, MLVSS continued to decrease to 5.45 ± 0.19 g/L at the early stage of granulation process in phase II while the mean particle size increased from 55 μm to 89 μm. The loss of sludge biomass might be attributed to the enhancement of up-flow velocity from 0.5 m/L to 1.2 m/L at day 61 to further promote anaerobic granulation.

### AHL level correlated with granulation

**Figure 2:**
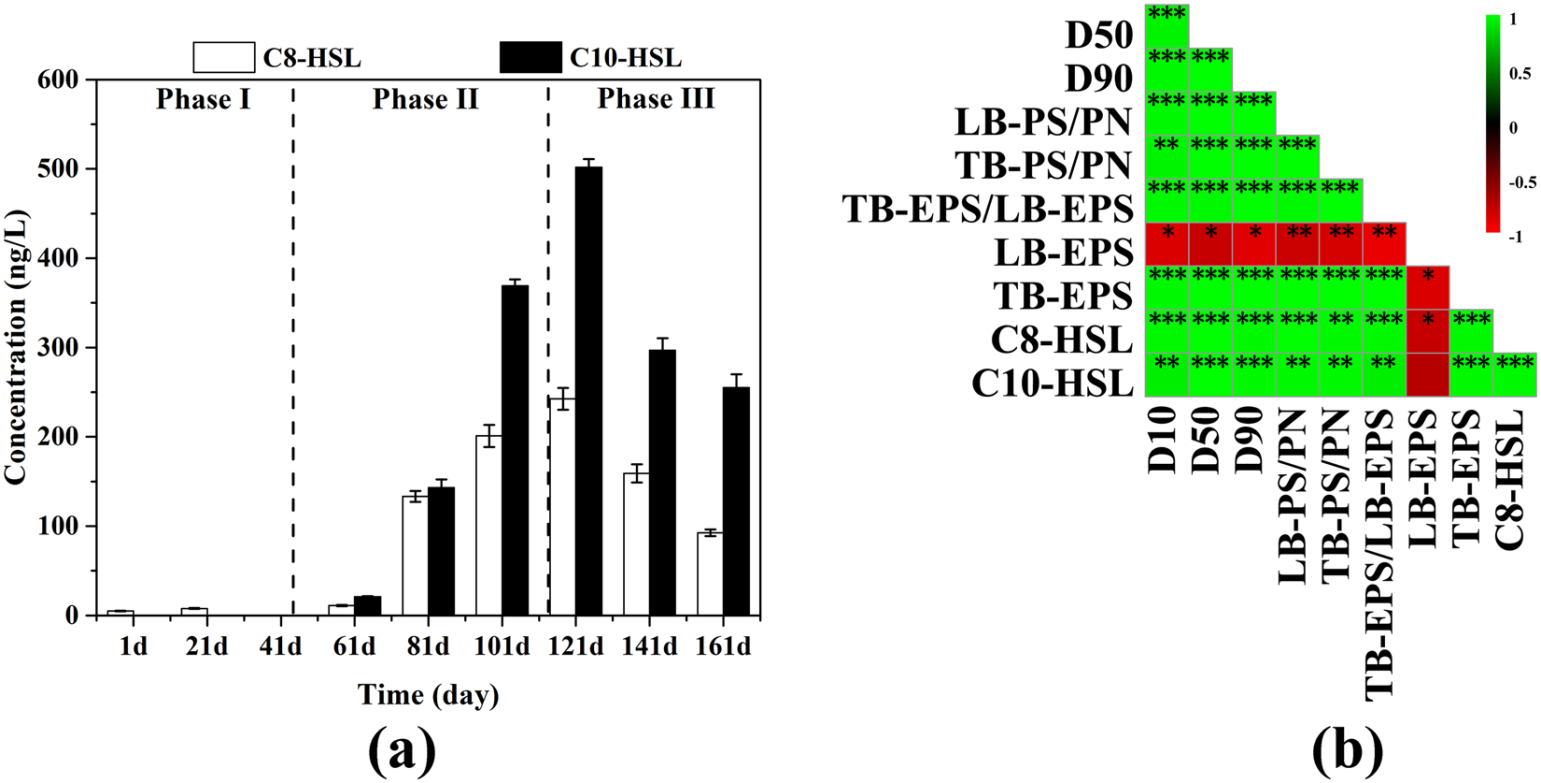
The AHLs concentration correlate with granulation and EPS production. (**a**) AHL was qualitatively and quantitatively determined by HPLC-MS/MS. Error bars represent standard deviation of three technical replicates. (**b**) The Pearson correlation among AHL, EPS and granule size. *P < 0.05, **P < 0.01 and ***P < 0.001 were used to indicate different significance.

On account of that the evaluation of AHL-mediated QS level almost relied on characterization of AHLs in anaerobic sludge phase^13^, qualitative and quantitative determination of AHLs in sludge phase was conducted using HPLC-MS/MS. Two kinds of AHLs including C8-HSL and C10-HSL were detected through the granulation process and therein C10-HSL had a higher concentration (Fig. 2a). These two AHL were commonly found in some other mature granules^13^. C10-HSL was under the detection limit (0.01 μg/L for the concentrated methanol extract) in phase I. In addition, the concentration of C8-HSL was below 7.81 ± 0.51 ng/L. Then C10-HSL emerged and increased drastically to 369 ± 7 ng/L during phase II, corresponding to the granule development stage. In the mean time, C8-HSL concentration increased over 20-fold during phase II in comparison to phase I. At the beginning point of phase III, the concentration of both C8-HSL and C10-HSL reached the highest value (243 ± 12 ng/L and 502 ± 9 ng/L, respectively), followed by subsequent significant gradual deduction. The concentration of AHL signals here was not only much lower than that in pure culture^26^, but also lower than that found in anammox culture^5^ and aerobic granular sludge^14^. But AHL based QS community behavior was also reported to be active at signal concentration as low as 20 pM in soil^27^. It was probable that AHL-mediated QS system might be active at different level in different ecosystem.

Both AHLs strongly and positively correlated with mean granule size during phase I-II transition (r > 0.9233, P < 0.003), indicated by the Pearson correlation analysis (Fig. 2b). However, no significant relationship between the AHLs and granulation was observed during phase III (Fig. S1). It was reported that AHL was important in maintaining granular structure^28^. However, it seemed that the effective AHL level for the maintenance of anaerobic granule might not have to be as high as that in granulation process as indicated by the decline of AHL level during the maturation stage (phase III). Although the role of AHL in granulation was unclear, this study clearly established a significantly strong positive correlation between AHL level and formation of anaerobic granules during phase I-II. It was thus tempting to speculate that AHL-mediated QS signaling system might participate in coordinating population behavior relevant to anaerobic granulation process.

### EPS production correlated with AHL concentration and granulation

**Figure 3:**
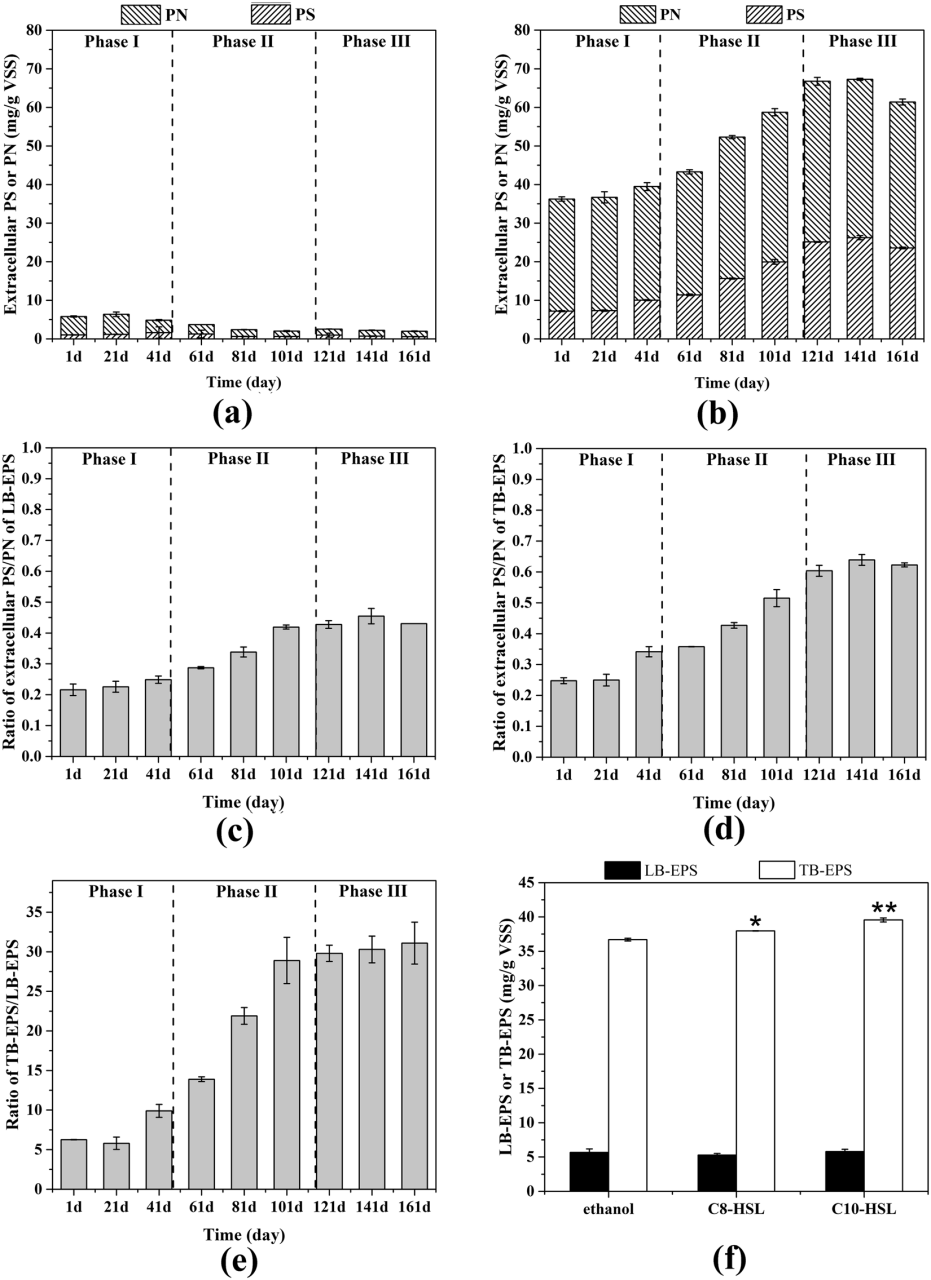
The concentration of LB-EPS (**a**) and TB-EPS (**b**) across the granulation process. The PS to PN ration of LB-EPS (**c**) and TB-EPS (**d**) and TB-EPS to LB-EPS ratio (**e**) during the operation period. Error bars represent standard deviation of three technical replicates. (**f**) Add-back of 5000 nM C8-HSL and C10-HSL increased the EPS production of anaerobic sludge. Two-way ANOVA was conducted and Bonferroni post-tests were performed to compute the significance. *P < 0.05, **P < 0.01 and ***P < 0.001 were used to indicate different significance.

EPS is considered to be crucial in reconstructing the three dimensional structure for biofilm and granules^29^. Also, EPS matrix allows cooperation and communication among cells in microbial aggregates^30^. According to the spatial distribution, EPS was divided into two parts, including the loosely bound EPS (LB-EPS) in the outer layer and the tightly bound EPS (TB-EPS) in the inner layer^31^. Both the PS and PN component was measured to determine the amount of LB-EPS and TB-EPS. The LB-EPS production decreased during the conversion from floccular sludge to granular sludge (phase I-II) while TB-EPS increased in concentration (Fig. 3a,b). As a result, the ratio of TB-EPS/LB-EPS increased significantly along with the transition from phase I to phase II (7.3 ± 2.2 vs 21.6 ± 7.5, *P* = 0.035) (Fig. 3e). Li and Yang^32^ suggested that more LB-EPS amount was associated with poorer cell attachment ability and relatively weak microbial aggregate structure. The improvement of ratio of TB-EPS/LB-EPS was assumed to contribute to formation of compact granules. Consistent with the TB-EPS profile, the ratio of PS/PN of both LB-EPS and TB-EPS increased during phase I-II transition (Fig. 3c,d). The synthesis of PS could influence cell adhesion ability and be conducive to the formation and stability of microbial aggregates^33^. The growth of PS/PN ratio accompanied by granulation process was also observed in some other studies, implying that higher proportion of PS in EPS was beneficial to cellular aggregation^14,34^.

Except LB-EPS, all the EPS properties, including TB-EPS concentration, PS/PN ratio and TB-EPS/LB-EPS, exhibited significant strong positive correlation (r > 0.9429, *P* < 0.01)with AHLs and granulation during phase I-II (Fig. 2b). On the contrary, there is no significant correlation among EPS property, AHL and particle size during the maturation stage (phase III) (Fig. S1). Previous studies have demonstrated that AHL based QS regulatory is responsible for EPS synthesis and biofilm formation in some pure culture system^35,36^. In terms of complex engineered habitats with high microbial diversity, high EPS production were positively correlated to high AHL concentration^4,11,37^. The highly strong positive correlation among EPS production, AHL level and granulation implied an interrelationship of these three processes. It was impossible to figure out the cause and consequence based on the Pearson analysis, especially in such a complex anaerobic microbial community of thousands of microorganisms. However, the EPS production increased after exogenous addition of AHLs to floccular sludge in the add-back experiment (Fig. 3f). The exogenous addition of C8-HSL and C10-HSL significantly increased the TB-EPS production by 3.5% and 7.8%, respectively, but minor difference was found in LB-EPS amount in comparison to that in negative control. This phenomenon might be in support of the speculation that the AHL-mediated QS system played a role in EPS regulation. The enhanced EPS production could not be attributed to the selection pressure of exogenous AHL on species on account of the short experiment time^14^. Also, the increase in EPS amount was impossible to be a consequence of exogenous AHL acting as nutritional source for microorganisms because the concentration of exogenous AHL was as low as 5000 nM^38^. It was interesting to notice that the PS to PN ratio showed a strong positive correlation with AHL level during phase I-II (Fig. 2b). Li and Zhu^37^ reported that AHL based QS participated in changing the composition of EPS in the formation of biofilm. The combination of these observations suggested that the AHL-based QS signaling was probably relevant to not only the regulation of EPS synthetic amount but also regulating the component proportion of EPS to facilitate anaerobic granulation process.

### Sludge community correlated with AHL concentration and granulation

**Figure 4:**
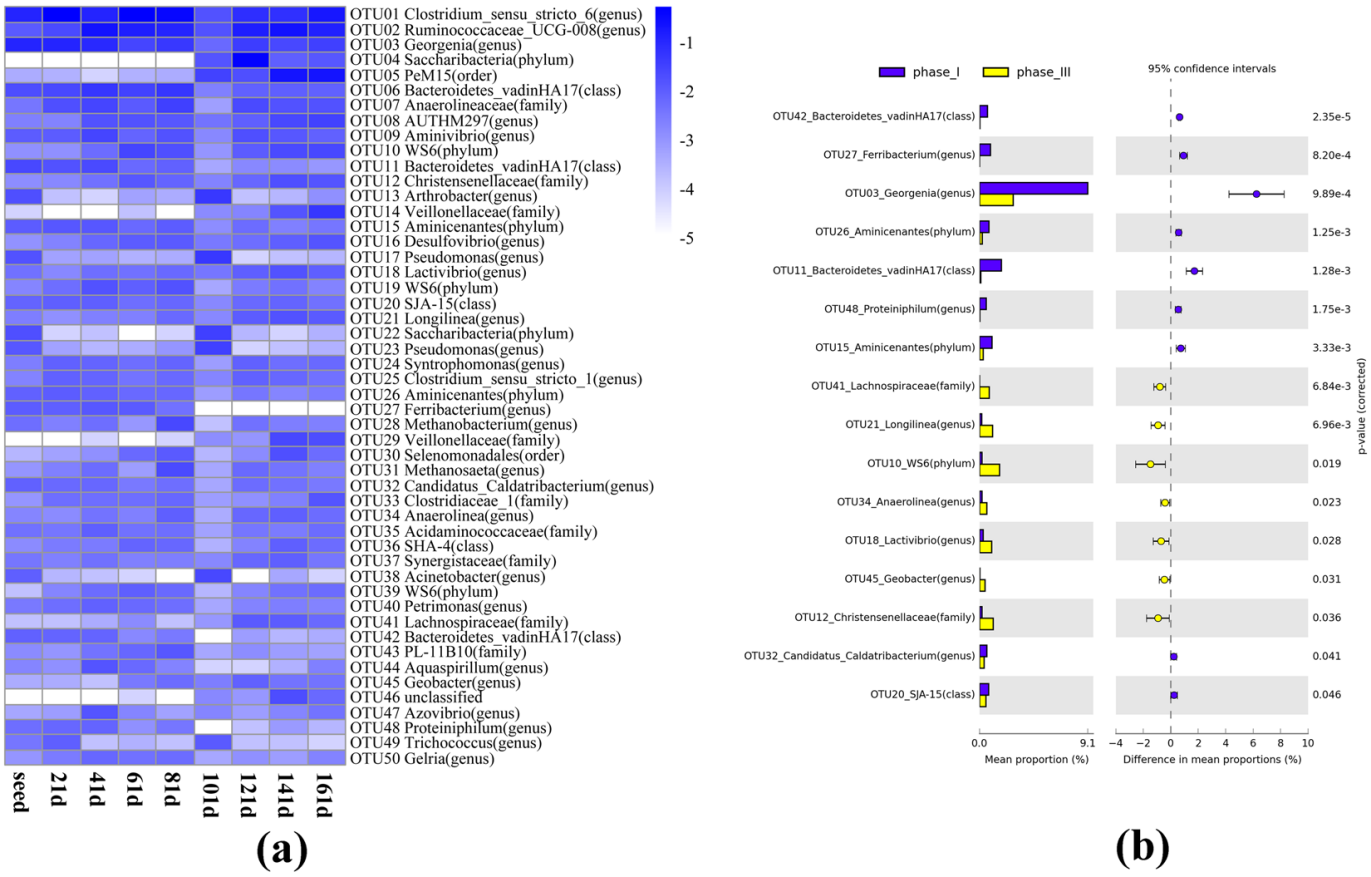
(**a**) The proportion of the 50 most abundant microorganisms during the operation period. A color gradient was used to represent the abundance value after log10 transformation. (**b**) Average abundance of OTUs that were differently abundant in phase I and phase III, colored in blue and yellow, respectively.

The 16S rRNA gene amplicons of all the samples were sequenced on Miseq platform to generate at least 14545 filtered sequences of each sample, so all the datasets were normalized to 14545 sequences for downstream data analysis. Then all the sequences were clustered into operational taxonomic units (OTUs) at 3% cutoff. The top two abundant OTUs that were both classified into order Clostridiales accounted for 26.0% of the total community (Fig. 4a). The Clostridiales was a well-known fermenter and functionally important in anaerobic digesters^39–41^, but there was no obvious change in the proportion of Clostridiales when the floccular sludge transformed into granule. The most remarkable variation in the microbial community composition during the granulation process was the significant reduction in Georgenia abundance (Fig. 4b, phase I vs phase III). Georgenia was applied in the decolourization and degradation of red dye in a previous study^42^, but it appeared to be humbly favourable in anaerobic granules here.

**Figure 5:**
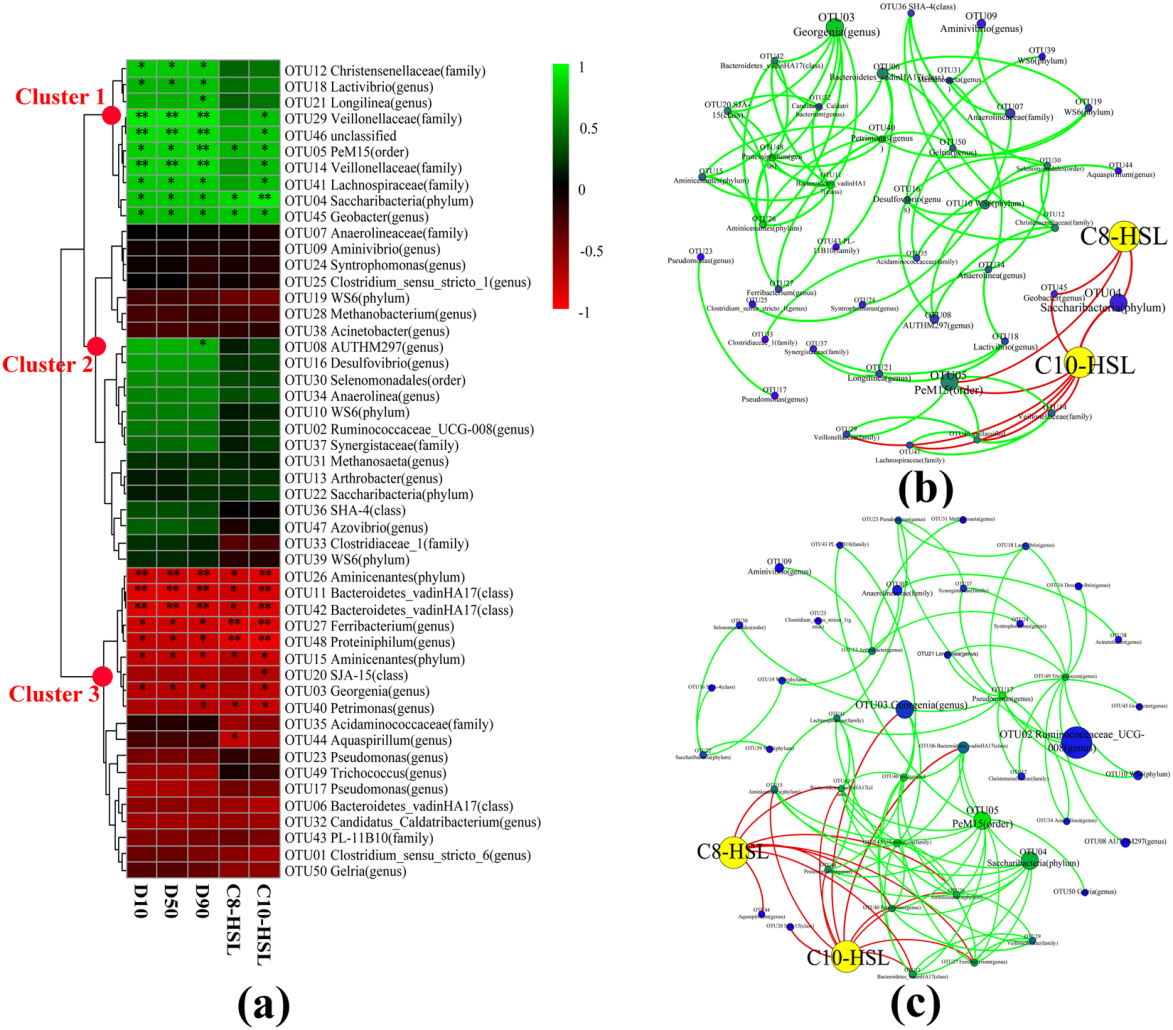
(**a**) Clustering of the 50 most abundant microorganisms in relation to AHLs level and granulation. Spearman’s correlation analysis was performed to determine the correlations between the abundance of top 50 microorganisms and AHL concentrations, granulation. *P < 0.05, **P < 0.01 and ***P < 0.001 were used to indicate different significance. Positive (**b**) and negative (**c**) co-occurrence patterns of the relationships among top 50 OTUs and AHLs. An edge standed for a strong (Spearmans’s ρ > 0.8 or ρ < −0.8) and significant (*P* < 0.05) correlation. The nodes of AHLs were colored in yellow. The edges between AHLs and OTUs were colored in red while the edges between different OTUs were colored in green.

The relationships between the top 50 abundant OTUs and AHL concentration and sludge particle size across all the 168 days of operation were determined based on Spearman’s correlation coefficients calculation. The 50 microorganisms were clustered into three clusters, referred to as Cluster I, II and III, using average method (Fig. 5a). Cluster I microorganisms positively correlated with AHLs concentration and granulation whereas Cluster III microbes showed negative correlation with AHLs and granulation. Community members in Cluster II demonstrated no correlation to AHL. As indicated by the strong positive correlation with AHL, the QS positively relevant community members in Cluster I might either be AHL producers or benefit from QS^14^. To our knowledge, the identified AHL-producers till now were all classified into alpha, beta and gamma Proteobacteria^22,29^. Among the 10 microorganisms in Cluster I, only one tag assigned to genera Geobacter was affiliated to Proteobacteria and held forth the possibility of being an AHL-producer. To our knowledge, there has been only one known anaerobic microorganism named *Pseudomonas aeruginosa* was previously confirmed to be related to AHL-based QS system^43^. Although the other Cluster I members could not secrete AHL signals, they were highly probable to participate in the expression of specific gene via responding to AHL-based QS system. A genome survey by Case, *et al*.^44^ suggested that 45 out of 512 complete genomes contained homologues of the AHL receptor gene but not AHL synthase gene. As the 10 members in Cluster I were all positively related to the formation of anaerobic granules, the AHL regulated genes might be involved in the granulation process. The low abundance of potential AHL-producer was consistent with a previous report suggesting that highly complex community was dominated by AHL-quenching or AHL-irrelevant bacteria^45^. Although community members in Cluster I only accounted for 13.0% of the total sequences, the proportion of Cluster I microorganisms significantly increased from 0.8 ± 0.3% in phase I to 34.9 ± 8.2% in phase III (*P* < 0.03) (Table S1). A similar shift in the community structure was also observed in aerobic granulation process^22^. The growing percentage of QS positively relevant microbes during the transition from floccular sludge to granular sludge indicated that AHL based QS system might be involved in restructuring community composition to promote granulation process. Although it was unclear whether AHL based signaling mechanism was a direct cause of granulation, this study established a strong positive relationship among QS signaling, EPS production, 10 microorganisms in Cluster I and granulation process. These strong correlations gave a suggestive hint that AHL might play an important role in coordinating the highly diverse community to aggregate into compact granules, potentially via regulating the expression of EPS-relevant genes in the 10 microorganisms of Cluster I (Fig. S2).

Network analysis enables the visualization of co-occurrence pattern of microbes and has been successfully applied to look into the interrelationships among soil communities^46,47^. The co-occurrence analysis was accomplished by comparing the abundance of top 50 OTUs and AHL concentrations with each other and a robust correlation was confirmed if Spearman’s coefficient >0.8 and *P* < 0.05. The resulting network comprised of 81 robust positive correlations and 96 robust negative correlations (Fig. 5b,c), implying highly effective cooperation and competition activity among different microorganisms. A total of 18 negative correlations were determined between the AHLs and microbial members while only 10 positive correlations were identified. It was worth noting that C10-HSL had 7 positive correlation with microorganisms, more than C8-HSL did (only 3 positive correlations) (Fig. 5b). More robust positive correlations between C10-HSL and different bacteria might support the hypothesis C10-HSL based QS signaling was more active in granulation process than the C8-HSL-mediated QS. This was in accordance with the result of add-back experiment that exogenous addition of C10-HSL significantly increased TB-EPS amount by 7.8%, larger than 3.5% with exogenous C8-HSL. The combination of these two observations implied that exogenous addition of C10-HSL might be a promising strategy to accelerate anaerobic granulation process.

## Conclusion

During the conversion from floccular sludge to granular sludge (phase I-II), two kinds of AHLs including C8-HSL and C10-HSL had been identified and strongly positively correlated to granulation process. As indicated by add-back experiment and network analysis, exogenous C10-HSL might contribute to promoting anaerobic granulation greater than C8-HSL. The granulation process was accompanied by an increase in the abundance of QS-relevant microorganisms. The strong positive correlation among AHL concentration, EPS (except LB-EPS), granulation and microbial community implied that AHL based QS signaling might be involved in mediating community behavior related to granulation, potentially through regulating the production and composition of EPS. This study helps to look deeply into the biological fundamentals of AHL-mediated QS signaling in anaerobic granulation process and sheds light on the future application of AHL-based strategy to promote anaerobic granulation.

## Electronic supplementary material

Supplementary material

## Data Availability

The data within my uploaded manuscript file is available.
