# Supplementary material for: The biological role of N-acyl-homoserine lactone-based quorum sensing (QS) in EPS production and microbial community assembly during anaerobic granulation process
Source: Sci Rep. 2018 Oct 25;8:15793. doi: 10.1038/s41598-018-34183-3 (PMC6202354; doi:10.1038/s41598-018-34183-3)
Supplement: Supplementary file 1 — Supplementary material [file 41598_2018_34183_MOESM1_ESM.pdf]

Supplementary material

The biological role of N-acyl-homoserine lactone-based quorum sensing (QS) in EPS production and microbial community assembly during anaerobic granulation process

Haijun Ma, Sijia Ma, Haidong Hu, Lili Ding, Hongqiang Ren*

State Key Laboratory of Pollution Control and Resource Reuse, School of the Environment, Nanjing University, Nanjing 210023, Jiangsu, PR China

*Corresponding author: Hongqiang Ren; Tel: +86 25 89680512;
fax: +86 25 89680569. E-mail address: hqren@nju.edu.cn

Materials and methods

Bioinformatics analysis The raw read1 and read2 datasets were demultiplexed by trimming the barcode sequences with no more than 1 mismatch. Then the sequences with the same ID were picked from the remaining read1 and read2 datasets by a self-written python script. Bases with average quality score lower than 25 over a 25 bases sliding window were excluded and sequences which contained any ambiguous base or had a final length shorter than 200 bases were abandoned using Sickle [1]. The paired reads were assembled into contigs and any contigs with an ambiguous base, more than 8 homopolymeric bases and fewer than 10 bp overlaps were culled. After that, the contigs were further trimmed to get rid of the contigs that have more than 1 forward primer mismatch and 2 reverse primer mismatch. The primer sequences were trimmed off. Then the sequences that were the same with each other were merged as one unique sequence to accelerate the filtering calculation. The remaining unique sequences were aligned using SILVA bacterial reference database. Afterwards, the sequences that did not align to the correct region were excluded [2]. Both ends of the sequences were trimmed to ensure that all the sequences started and ended at the same position. Additionally, a preclustering algorithm was employed to merge sequences by allowing 1 bp difference for every 100 bp [3]. Chimeras were screened and removed from the resulting sequences using Uchime packaged in Mothur [4]. All the datasets were normalized to 14545 sequences by the “sub.sample” command. The filtered sequences were classified against the

SILVA 16S reference database (version 128) using a naïve Bayesian classifier built in Mothur with an 80% confidence score [5]. Sequences passing through all the filtration were also clustered into OTUs at 3% dissimilarity level. Then a “classify.otu” function was utilized to assign the phylogenetic information to each OTU.

Supplementary References

1. Joshi NA, FJ. 2011. Sickle: A sliding-window, adaptive, quality-based trimming tool for FastQ files (Version 1.33) [Software].

2. Schloss PD. 2010. The Effects of Alignment Quality, Distance Calculation Method, Sequence Filtering, and Region on the Analysis of 16S rRNA Gene-Based Studies. *Plos Comput. Biol.* **6**: e1000844.

3. Kozich JJ, Westcott SL, Baxter NT, Highlander SK, Schloss PD. 2013. Development of a Dual-Index Sequencing Strategy and Curation Pipeline for Analyzing Amplicon Sequence Data on the MiSeq Illumina Sequencing Platform. *Appl. Environ. Microb.* **79**: 5112-5120.

4. Edgar RC, Haas BJ, Clemente JC, Quince C, Knight R. 2011. UCHIME improves sensitivity and speed of chimera detection. *Bioinformatics* **27**: 2194-2200.

5. Mizrahi-Man O, Davenport ER, Gilad Y. 2013. Taxonomic Classification of Bacterial 16S rRNA Genes Using Short Sequencing Reads: Evaluation of Effective Study Designs. *Plos One.* **8**: e53608.

Table S1. The proportion of microorganisms of each cluster in different phases

	Phase I	Phase II	Phase III
Cluster1	0.8±0.3%	3.2±1.8%	34.9±8.2%
Cluster2	17.3±15.2%	21.2±8.2%	27.8±7.1%
cluster3	44.5±19.8%	37.3±23.4%	15.8±6.1%

Figure caption

Fig. S1. The relationships among AHLs concentration, granulation and EPS production during phase III. The relationships during phase III were shown with a color gradient denoting Pearson correlation coefficients. Granulation was determined by particle size distribution (D10, D50 and D90). Significant differences were indicated as follows: $*P < 0.05$, $**P < 0.01$.

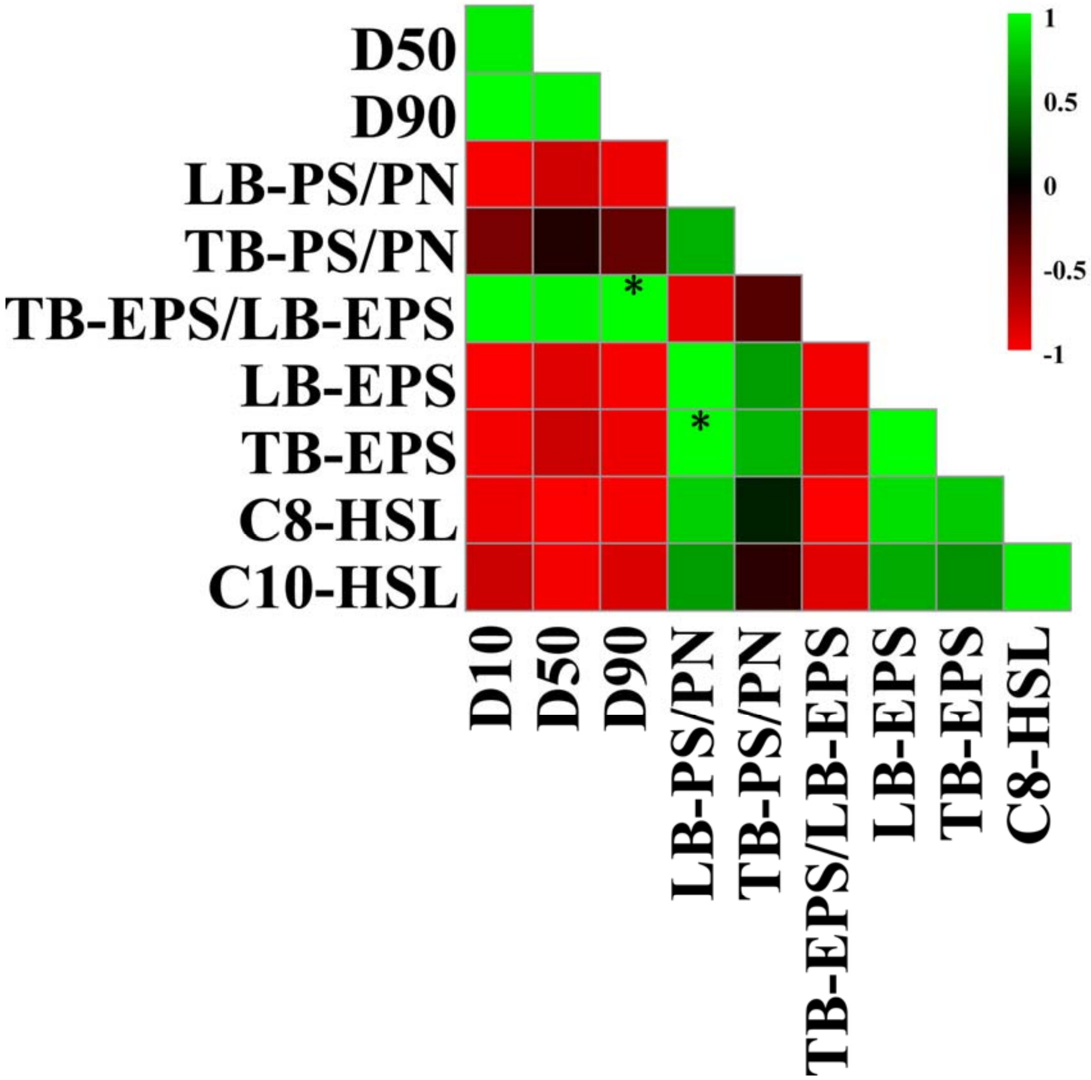

Fig. S1. The relationships among AHLs concentration, granulation and EPS production during phase III. The relationships during phase III were shown with a color gradient denoting Pearson correlation coefficients. Granulation was determined by particle size distribution (D10, D50 and D90). Significant differences were indicated as follows: **P* < 0.05, ***P* < 0.01.
